# The relationship between the aspects of connectedness and sustainable consumption

**DOI:** 10.3389/fpsyg.2023.1216944

**Published:** 2024-01-16

**Authors:** Petra Jansen, Sabine Hoja, Martina Rahe

**Affiliations:** ^1^Faculty of Human Sciences, University of Regensburg, Regensburg, Germany; ^2^Institute of Psychology, University of Koblenz, Koblenz, Germany

**Keywords:** inner transformation, sustainable behavior, connectedness, self-love, connectedness to nature, prosocialness

## Abstract

Internal transformative qualities are essential contributing factors to sustainable behavior. Besides awareness, insight, purpose, and agency, connectedness is one of those inner qualities. In this study, we investigated the relationship between connectedness to oneself (self-love), towards the environment (connectedness to nature), towards other human beings (pro-socialness), and sustainable behavior towards clothes and food. One hundred thirty-nine mostly students participated. The results showed that self-love, connectedness to nature, and pro-socialness correlate. Sustainability behavior towards food was predicted by pro-socialness, the choice of diet, and environmental and ethical reasons for nutrition. Sustainable behavior towards clothes was predicted by connectedness to nature. This study hints that the factors of inner transformative qualities and the type of sustainable behavior must be investigated differently. It strengthens the multi-facet dimensions of sustainable behavior.

## 1 Introduction

In the research on sustainable consumption, many studies concentrate on analyzing a single behavior (Geiger et al., 2018b), which leads to a fragmented picture. To overcome this lack and to provide a valid measurement of consumer behavior, Geiger et al. (2018a) developed a three-dimensional consumption model with the dimensions of consumption phases (e.g., acquisition and usage phase), consumption areas (e.g., mobility, food, clothes), and sustainability spheres (e.g., socioeconomic sphere).

In recent years, some research has investigated which internal factors, besides more external factors like norms and attitudes, contribute to sustainable consumption behavior (Ives et al., 2020). Wamsler et al. (2021) defined five clusters of internal factors, the so called inner transformative qualities, that can be seen in sustainable consumption behavior: Awareness, connection, insight, purpose, and agency. In this study, the connection aspect will be investigated in depth. The feeling of connectedness will be investigated by the aspects of being connected to oneself (self-love), being attached to other human beings (pro-socialness), and connectedness to the environment (connectedness to nature).

### 1.1 Self-love: the connectedness to oneself

By the concept of self-love, the connectedness to oneself can be investigated. As described in Henschke (2022), self-love is a controversial construct. On the one hand, it is desirable because it is related to well-being and is a crucial resilience factor in preventing mental illness (Solimar, 1987). On the other hand, it is often confused with narcissism (Henschke, 2022). However, Fromm (1939) already differentiates between self-love and narcissism; self-love is disguised as self-loathing (Kowalchyk et al., 2021). Even though the literature on self-love has grown for the mainstream, scientific research is rare, and a definition seems to be still missing; if self-love is printed in the title, self-esteem or narcissism are often investigated. By doing an inductive thematic analysis, Henschke and Sedlmeier (2021) found that self-contact (perception and encountering of oneself), self-acceptance (accepting one’s own shadow and strengths) and self-care (treating oneself and shaping relationships) are the three essential constructs of self-love.

### 1.2 Pro-socialness: the connectedness to others

Connectedness to others can be investigated through pro-socialness. Pro-socialness or prosocial behavior describes the behavior through which people benefit others (Eisenberg, 1982). It can be distinguished between an emotional response to another person’s suffering and a cognitive reaction, such as the ability to take another person’s perspective (Eisenberg et al., 2007). Prosocial behavior can be differentiated into altruistically motivated, norm-motivated, and self-reported prosocial behavior, which can be improved by specific mental training (Böckler et al., 2018). The complexity of this phenomenon is also expressed in the diversity of the measurements, which range from self-reports and game theoretical paradigms to computerized interactions which resemble real-life scenarios (Böckler et al., 2018). Prosocial behavior can be increased, for example, by self-reflection (Lewis et al., 2021) and mindfulness training (Berry et al., 2020).

### 1.3 The connectedness towards nature

Connectedness to nature can be considered as a stable state which is reflected by a sustained awareness of the interrelatedness between the own person and the rest of nature (Thiermann and Sheate, 2021). However, also other explanations exist: Mayer and Frantz (2004) suggest it as a trait that enables the individual to feel emotionally connected to the natural world. The term nature relatedness is also used (Nisbet et al., 2009), including the awareness of all nature aspects. Key elements are the expansion of self-identity including the natural environment and the experience of belonging to nature (Whitburn et al., 2020). Connectedness to nature is positively related to several outcomes as it is, for example, well-being (Mayer and Frantz, 2004), health (Nisbet and Zelenski, 2013), and happiness (Nisbet and Zelenski, 2013).

### 1.4 The relationship between connectedness and sustainable consumption behavior

First of all, it has been shown that connectedness to nature is correlated to self-love and pro-social behavior (Rahe and Jansen, 2023). Even though in that study (Rahe and Jansen, 2023), a correlation between self-love and pro-social behavior was not given, one other study found positive relations between self-care and altruistic behavior (Corral-Verdugo et al., 2021). Beside this, there is evidence that connectedness to nature (Whitburn et al., 2020) and prosocial behavior are often linked to sustainable behavior (de Groot and Thøgersen, 2018). This relationship can be explained in the framework of the two-pathway model of pro-environmental behavior (Thiermann and Sheate, 2021), which includes—next to the normative pathway built by the relevance of social and personal norms—a relational pathway based on connectedness to nature, empathy, and compassion: If someone increases the relational pathway through mindfulness practice, the motivation to act pro-environmentally becomes more internalized (Thiermann et al., 2020). To conclude, the three aspects of connectedness, self-love, pro-socialness, and connectedness to nature, can be seen as elements of the relational paths and should predict sustainable behavior.

Sustainable behavior was investigated in the framework of the three-dimensional consumption model of Geiger et al. (2018a) and especially for the sustainable consumption of food, clothes, and the general consumption behavior. The consumption areas of housing and mobility (Geiger et al., 2018a) were not included because they are very much income-related, and the results could be biased by mainly students taking part in this study. It has also been shown that younger participants have a higher willingness to pay a higher price for sustainable clothing (Dangelico et al., 2022) and sustainable food consumption in Germany (Paslakis et al., 2020) than older ones.

### 1.5 Goal of the study

The study’s main goal was to investigate the relationship between one internal transformative quality, the one of connectedness, and sustainable consumption behavior toward food, cloths and in general. This adds to the proposed models of Wamsler et al. (2021) and Thiermann and Sheate (2021). The following hypotheses were investigated in detail:

1. There is a correlation between the measurements of self-love, pro-socialness, connectedness to nature (Corral-Verdugo et al., 2021; Rahe and Jansen, 2023) and due to the two-path model of pro-environmental behavior (Thiermann et al., 2020) and the relevance of internal transformative qualities (Wamsler et al., 2021) to sustainable consumption behavior.
2. In addition to the correlational analysis, we assume that the three aspects of connectedness predict the measurement of the three elements of sustainable consumption behavior investigated here. For measuring the consumption behavior towards food, the preferred diet will be included as another predictor and exploratorily, the importance of nutrition and ethical and health reasons for the choice of nutrition.
3. We hypothesize that the relationship between self-love and the three aspects of pro-environmental consumption behavior should be mediated via pro-socialness and connectedness to nature. There is evidence that connectedness to nature (Whitburn et al., 2020) and prosocial behavior (de Groot and Thøgersen, 2018) are often linked to sustainable behavior and that self-love with the dimension of self-care plays an important role (Corral-Verdugo et al., 2021).

## 2 Method

### 2.1 Participants

In this study, 139 participants (68 men, 69 women, 2 diverse) between 19 and 56 years (M = 22.65, SD = 3.73) took part. Fifty of them were vegans/vegetarians, and 89 were omnivores, see an overview in Table 1. The power analyses (Faul et al., 2007, see Supplementary material) showed that at least 127 participants were needed. Participants were recruited from the Faculty of Human Sciences of the University of Regensburg and social media.

**Table 1:** Demographic data.

	Sex	Age	Education state	Netto income	Importance of nutrition^a^	Diet due to environmental/ethical reasons^a^	Diet due to health reasons^a^	Active meditation (min/week)	Mindful movement experience (min/week)
**Vegetarian/vegan** **(*N* = 50)**	Female: 66% Male: 30% Other: 4%	22.74 (2.36)	No qualification: 0% “Mittlere Reife”: 0.0% A-levels: 96.0% Bachelor: 2.0% Master: 2.0%	–1000€: 82.0% –2000€: 10.0% –3000€: 0.0% –4000€: 0.0% > 4000€: 0.0% Not specified: 8.0%	1: 2.0% 2: 0.0% 3: 12.0% 4: 44.0% 5: 42.0% M = 4.24 (0.82)	1: 0.0% 2: 4.0% 3: 12.0% 4: 52.0% 5: 32.0% M = 4.12 (0.77)	1: 0.0% 2: 2.0% 3: 10.0% 4: 50.0% 5: 38.0% M = 4.24 (0.72)	22.22 (48.64)	34.50 (39.13)
**Omnivorous** **(*N* = 89)**	Female: 59.6% Male: 40.4% Other:0.0%	22.61 (4.32)	No qualification: 1.1% “Mittlere Reife”: 1.1% A-levels: 92.1% Bachelor: 2.2% Master: 3.4%	–1000€: 78.7% –2000€: 12.4% –3000€: 3.4% –4000€: 0.0% > 4000€: 0.0% Not specified: 5.6%	1: 0.0% 2: 1.1% 3: 13.5% 4: 62.9% 5: 22.5% M = 4.07 (0.64)	1: 2.2% 2: 14.6% 3: 40.4% 4: 38.2% 5: 4.5% M = 3.28 (0.85)	1: 0.0% 2: 2.0% 3: 12.4% 4: 59.6% 5: 28.1% M = 4.16 (0.62)	9.84 (25.3)	22.71 (40.52)

Mittlere Reife = school leaving certificate after 10 years of school.

a Categories: 1 = not important, 2 = not very important, 3 = neutral, 4 = important, 5 = very important.

### 2.2 Material

This study investigated a demographic questionnaire, the questionnaires of self-love, pro-socialness, connectedness to nature, and the measurements of different aspects of sustainable consumption behavior.

*Demographic questions* (see Siebertz et al., 2022). First of all, a demographic questionnaire was used with the following variables: Sex (male, female, diverse), age, education state (categorial: high school, Abitur, bachelor, master, PhD), netto income [categorial (€ per month): up to 1000€, 1001–2000€, 2001–3000€, 3001–4000€, more than 4000€, no answer], frequency of active meditation experience (never, in minutes per year, month, week or day) and frequency of mindful movement experience (yoga, TaiChi, etc.) (never, in minutes per year, month, week or day), choice of diet (1 = vegetarian/vegan, 2 = omnivore), the importance of nutrition (1 = not at all to 5 = very much), the importance of the choice of a diet due to environmental and ethical reasons (1 = not important at all to 5 = very important), the importance of the choice of a diet due to health reasons (1 = not important at all to 5 = very important).

*Self-love* (Henschke, 2022). Self-love was investigated with the self-love questionnaire, which included 27 items and had to be answered on a 5-point scale from 1 = not true at all to 5 = entirely true. The confirmatory factor analysis (Henschke, 2022, subsample 1, *N* = 483) indicated good fit: χ^2^-test: χ^2^ (312) = 717.57, CFI = 0.94, RMSEA = 0.052, SRMR = 0.05. Construct validity was good. The use of the scale in this study showed excellent internal consistency (Cronbach’s alpha = 0.920).

*Prosocialness Scale for Adults* (Caprara et al., 2005). 16 items measured prosocial behavior answered on a 5-point scale ranging from 1 = never/rarely true to 5 = almost always/always true. An example item was “I try to console those who are sad.” The questionnaire was based on item response theory (IRT). Reliability (α = 0.91), difficulty parameter, and discrimination parameter were suitable, and the IRT analyses support effectiveness and sensitivity (Caprara et al., 2005). For the German version, the questionnaire was forward and backward translated. One item had to be removed (“I am available for volunteer activities to help others”) because of low corrected item-total correlations (< 0.3). For the remaining 15 items, internal consistency was good (Cronbach’s alpha = 0.836).

*Connectedness to Nature Scale* (CNS, Pasca et al., 2017). Connectedness to nature was measured with 13 items, which were answered on a 5-point scale ranging from 1 = strongly disagree to 5 = strongly agree. An example item was “Like a tree can be part of a forest, I feel embedded within the broader natural world.” For the German version, the questionnaire was forward and backward translated. Two items had to be removed because of low corrected item-total correlations. Internal consistency was good for the remaining eleven items (Cronbach’s alpha = 0.840).

*Measurement of sustainable consumption* (Geiger et al., 2018a). For the measurement of sustainable consumption behavior (SCB) towards food, clothes, and in general, the questionnaire of Geiger et al. (2017) was used. This means that SCB was assessed with self-reports on behavior in three different areas, food with 16 items, clothing (with 16 items), and in general (with six items). The scale is based on the cube model of SCB (Geiger et al., 2018a). Answers were given on a seven-point scale. For food, we eliminated eight items because of low corrected item-total correlations. This resulted in a scale with acceptable internal consistency (Cronbach’s alpha = 0.730). For clothes, four items were eliminated for the same reason. For the remaining scale, internal consistency was good (Cronbach’s alpha = 0.810). No reliable scale could be calculated for the subscale “in general” because the items seemed very heterogeneous, with low correlations between them.

### 2.3 Procedure

All questionnaires were implemented in SoSci Survey (Leiner, 2019). The link was advertised to the participants via newsletter and social media. First, participants gave informed consent and then provided demographic information. After this, they completed the questionnaires on self-love, pro-socialness, and connectedness to nature. Then the questionnaires regarding sustainability for food, clothes, and generally were applied. Afterwards, they were thanked for their participation.

The study was conducted according to the ethical guidelines of the Helsinki declaration and approved by the Ethic Research Board of the University (no. 22-3059-101). The study was preregistered at OSF.^1^

### 2.4 Statistical analysis

First, correlations (hypothesis 1) between the study variables were calculated. After this and following hypothesis 2, regression analysis with the criterion sustainable clothes was conducted with the three predictors self-love, pro-socialness, and connectedness of nature. A regression with the three predictors mentioned above and the factor “choice of diet” was conducted for the criterion of sustainable food consumption. Exploratorily, the variables “importance of nutrition,” “diet choice due to ethical reasons,” and “diet choice due to health reasons” were also integrated. Unlike pre-registration, no regression analysis could be calculated for the criterion of sustainable consumption in general because the scale was unreliable. The third hypothesis could not be analyzed because no significant correlations were found between self-love and sustainable consumption of food and clothes.

## 3 Results

To give a first overview of the data, correlations between the study variables and means and standard deviations are tabled in Table 2.

**Table 2:** Means and standard deviations of and correlations between the study variables.

		M	SD	1	2	3	4	5	6	7	8	9
1	Food	4.63	0.88	–								
2	Clothes	3.39	0.95	0.589^**^	–							
3	SL	3.75	0.51	0.001	−0.001	–						
4	PS	3.99	0.45	0.259^**^	0.209^*^	0.247^**^	–					
5	CN	3.12	0.74	0.189^*^	0.370^**^	0.263^**^	0.355^**^	–				
6	Age	22.65	3.73	0.043	0.098	−0.070	−0.058	0.079	–			
7	Choice of diet	1.64	0.48	−0.507^**^	−0.397^**^	0.171^*^	−0.102	−0.144	−0.017	–		
8	Importance of nutrition	4.13	0.71	0.226^**^	0.216^*^	0.122	−0.009	0.185^*^	−0.101	−0.117	–	
9	Environmental/ethical reasons	3.58	0.92	0.622^**^	0.437^**^	0.002	0.188^*^	0.298^**^	−0.070	−0.441^**^	0.217^*^	–
10	Health reasons	4.19	0.66	0.238^**^	0.108	0.176^*^	0.036	0.220^**^	−0.131	−0.061	0.680^**^	0.167^*^

SL: self-love; PS: pro-socialness; CN: connectedness to nature. Choice of diet: 1 = vegetarian/vegan, 2 = omnivore. *p < 0.05, **p < 0.01.

The regression analysis with the criterion sustainable behavior towards clothes and the three predictors of self-love, connectedness to nature, and pro-socialness revealed that only connectedness to nature, *β* = 0.364, *p* < 0.001, was a significant predictor of clothes consumption. All predictors explained 15.8% of the variance of clothes consumption, *R* = 0.397, *F*(3,135) = 8.440, *p* < 0.001. Besides connectedness to nature, self-love, *β* = −0.124, *p* = 0.138, and pro-socialness, *β* = 0.111, *p* = 0.198, could not explain any incremental variance, see Table 3.

**Table 3:** Prediction of sustainable cloth consumption.

Variable	*B*	95% CI for *B*		SE *B*	ß
		*LL*	*UL*		
Constant	1.883	0.361	3.406	0.770	
CN	0.463	0.246	0.680	0.110	0.346***
PS	0.231	−0.122	0.585	0.179	0.111
SL	−0.228	−0.531	0.074	0.153	−0.124

CN: connectedness to nature; PS: pro-socialness; SL: self-love. ***p < 0.001.

Adding self-love, connectedness to nature, pro-socialness, and diet choice as predictors of sustainable behavior towards food consumption, the regression analysis revealed diet choice, *β* = −0.485, *p* < 0.001, and pro-socialness, *β* = 0.187, *p* = 0.019, as significant predictors. All predictors explained 30.3% of the variance of food consumption, *R* = 0.551, *F*(4,134) = 14.572, *p* < 0.001. Besides diet choice and pro-socialness, self-love, *β* = 0.025, *p* = 0.747, and connectedness to nature, *β* = 0.046, *p* = 0.565, could not explain any incremental variance. Adding the importance of nutrition, the ethical reasons for the choice of nutrition and the health reasons as additional predictors, three significant predictors (ethical reasons, *β* = 0.461, *p* < 0.001, diet choice, *β* = −0.289, *p* < 0.001, and pro-socialness, *β* = 0.167, *p* = 0.016) and four non-significant predictors (self-love, *β* = 0.003, *p* = 0.969, connectedness to nature, *β* = −0.084, *p* = 0.237, importance of nutrition, *β* =0.005, *p* = 0.951, and health reasons, *β* = 0.152, *p* = 0.078) explained 49.8% of the variance of food consumption, *R* = 0.706, *F*(7,131) = 18.555, *p* < 0.001, see Table 4.

**Table 4:** Prediction of sustainable food consumption.

Variable	*B*	95% CI for *B*		SE *B*	ß
		*LL*	*UL*		
Model 1					
Constant	4.306	2.941	5.670	0.690	
CN	0.055	−0.133	0.242	0.095	0.046
PS	0.364	0.061	0.667	0.153	0.187*
SL	0.043	−0.221	0.308	0.134	0.025
Choice of diet	−0.888	−1.160	−0.616	0.137	−0.485***
Model 2					
Constant	2.026	0.623	3.428	0.709	
CN	−0.100	−0.267	0.067	0.084	−0.084
PS	0.325	0.062	0.588	0.133	0.167*
SL	0.005	−0.225	0.234	0.116	0.003
Choice of diet	−0.529	−0.785	−0.273	0.129	−0.289***
Importance of nutrition	0.007	−0.204	0.217	0.106	0.005
Environmental/ethical reasons	0.444	0.305	0.582	0.070	0.461***
Health reasons	0.204	−0.023	0.432	0.115	0.152

CN: connectedness to nature; PS: pro-socialness; SL: self-love. *p < 0.05, ***p < 0.001.

## 4 Discussion

In this study, the relation of connectedness, as one specific internal transformative quality of sustainable consumption behavior and the sustainable behavior towards clothes and food is investigated. The study results show the relationship between the three investigated connectedness factors described here, which is in line with our first hypothesis: Connectedness to nature, pro-socialness, and self-love were related. However, only connectedness to nature was one of the relevant predictors of sustainability towards clothes, and pro-socialness was one of the predictors of sustainability towards food consumption. These results confirm only partly our second hypotheses.

### 4.1 The relationship between the connection to nature and sustainable behavior towards clothes

Because the fashion industry significantly affects nature and the environment, the relationship between the connection to nature and sustainable behavior concerning clothes seems plausible. This is in line with the results that connection to nature as well as, for example, compassion and gratitude for nature, might play an essential role in pro-environmental behavior (Tam, 2013, 2022). A more positive feeling of a connection to nature can be related to a higher sense of sustainable consumption of clothes because there is an awareness of the resources the fashion industry needs. A sustainable consumption behavior regarding clothes can be seen in second-hand clothing. One factor that is related to second-hand clothing is mindful consumption. However, since mindfulness and connection to nature are linked (Jansen et al., under review), the relationship between connectedness to nature and second-hand clothing might also be worth investigating in more depth.

### 4.2 The relationship between pro-socialness and sustainable behavior towards food

Pro-socialness predicts sustainable behavior towards food, the choice of diet, and ethical and environmental reasons: Participants with higher values on pro-socialness, vegetarians and vegans, and those who chose diet due to ethical and environmental reasons demonstrate a more sustainable behavior towards food. The choice of a vegetarian or vegan diet for ethical and environmental causes is related to a study conducted in Germany, where the motivation for a vegan diet was mainly explained by animal-related motives (89.7%). Environment-related reasons were also relevant but the least important (46.8%) among the sample (Janssen et al., 2016). However, animal-motivated, and environmentally motivated vegetarians construct their diets to achieve more pro-social and moral goals than vegetarians who have chosen the diet due to health reasons (Rosenfeld, 2019). Furthermore, the results align with a study demonstrating that vegetarians are more pro-social than omnivores (Nezlek and Forestell, 2020). Besides, vegetarianism is associated with higher empathy (Holler et al., 2021).

### 4.3 Theoretical implications: the different impact of the internal transformative quality of connectedness

The study’s results strengthen the importance of the internal transformative qualities and the connectedness factor. Nevertheless, they also go beyond this and demonstrate that those factors must be regarded differently regarding the specific aspect of sustainable consumption behavior. The study of Betzler et al. (2022) supports our results in some way; for example, in their research, problem awareness predicted sustainable food consumption but not fashion consumption. Especially for sustainable food consumption taken together, this hints that analysing a single sustainable consumption behavior is inappropriate. In our study, the predicting factors differ when only a differentiation due to the consumption area was applied. One reason might be that consumers are more concerned about what they eat than what they wear, a display of cognitive dissonance (Joy and Pena, 2017). This strengthens the claim of Geiger et al. (2018a) to consider sustainable consumption behavior as a multi-factorial construct. Whereas for sustainable behavior towards clothes, the explained variance is low for the investigated factors, it is high for the prediction of sustainable behavior towards food. One reason for this might be that in the prediction of food, ethical and environmental reasons were integrated. Those reasons are related to personal norms and are included in the normative pathway for explaining sustainable behavior (Thiermann and Sheate, 2021).

Nevertheless, although the assumption that self-love is related to connectedness to nature and to connectedness to other human beings (pro-socialness) is confirmed, it does not relate to sustainable behavior. This hints that sustainable behavior can be pronounced if the self-focus is neglected and the focus on others gets more critical. As it is investigated and conceptualized here, self-love focuses more on the individual before shaping the relationship with others (Henschke and Sedlmeier, 2021). Self-love might be necessary for well-being (Rahe and Jansen, 2023) but for sustainable consumption behavior other internal transformative qualities seemed more relevant.

### 4.4 Practical implications

This study hints that internal transformative qualities are important concerning sustainable consumption behavior, especially those related to other beings and nature. This is important for the individual as well as for the community. For example, engagement in pro-social behavior enhances the mental quality and well-being of the individual through place attachment on the one side and, on the other, fosters the engagement to protect the place (Ramkissoon, 2022). This might even influence residents’ support for tourism development (Ramkissoon, 2023). To foster pro-environmental behavior, structural changes, as well as lessons on pro-socialness and connectedness to nature, are important. Education for sustainable development should integrate both aspects.

### 4.5 Limitations and future research

The study presented here has some limitations: It is a cross-sectional correlational study with mostly university students who have participated. Almost 80% had an income below 1000€ per month, which made it challenging to spend more money on sustainable clothes and food. The study could be repeated with a non-student sample and in different cultures than in Western Europe. Besides, the study is limited since only one specific aspect of the internal transformative qualities has been investigated in depth, the element of connection. To achieve a complete picture of sustainable behavior’s internal transformative attributes, the other four aspects mentioned in Wamsler et al. (2021) should be investigated in-depth, too. This would also provide more insight into the relational path in the model of Thiermann and Sheate (2020). Another limitation is related to the strength of the study. It is one of the first studies investigating internal transformative qualities towards different aspects of sustainable consumption behavior, which were theoretically founded (Geiger et al., 2018). However, the questionnaires to investigate those different aspects of sustainable behavior must be adapted because some items show low item correlations.

## 5 Conclusion

However, even though the study is limited by the factors mentioned above, it contributes to two critical topics: The relevance of investigating the internal transformative qualities instead of inner transformational factors in more depth and distinguishing between different aspects of sustainable behavior. The topic of cognitive dissonance in sustainable behavior research might be interesting.

## Data availability statement

The datasets presented in this study can be found in online repositories. The names of the repository/repositories and accession number(s) can be found here: https://osf.io/8kgta/.

## Ethics statement

The studies involving humans were approved by Ethic Research Board of the University Regensburg (no. 22-3059-101). The studies were conducted in accordance with the local legislation and institutional requirements. The participants provided their written informed consent to participate in this study.

## Author contributions

PJ: conceptualization, analyzing, writing, editing. SH: implementation, editing. MR: analyzing, editing. All authors contributed to the article and approved the submitted version.

## Footnotes

^1^https://osf.io/3qac8/?view_only=5bea0aa030a24020bd8a37ac95383075
