# Supplementary material for: The relationship between the aspects of connectedness and sustainable consumption
Source: Front Psychol. 2024 Jan 16;14:1216944. doi: 10.3389/fpsyg.2023.1216944 (PMC10824907; doi:10.3389/fpsyg.2023.1216944)
Supplement: Supplementary file 1 [file Data_Sheet_1.DOCX]

Power analysis

For hypothesis 1, medium effect sizes for the correlations (r =.3) between the three aspects of connectedness and the three sustainable behavioral categories are assumed. Due to multiple testing (12 correlations), p was Bonferroni corrected and set to < .0023. The power-analysis (power of 1-ß = 0.80) resulted in N = 127 participants (Faul et al., 2007). With a medium effect size of f2 = 0.15, an alpha-level of p = .05, a power of 1-ß = 0.80, and 4 possible predictors for the dependent variable sustainable behavior of clothes and general sustainable behavior, a power analysis for the linear regression resulted in N = 85 (Faul et al., 2007). If for the variable sustainable behavior of food, the variables “the importance of nutrition”, “the importance of ethical reasons for nutrition”, and “the importance of health reasons for nutrition” are integrated into the regression for the sustainable consumption behavior of food, 103 participants are needed. Exploratorily three mediation analyses will be conducted between self-love and the three measurements of sustainable consumption behavior, and the mediators of pro-socialness and connectedness to nature if the variables are correlated.
